# Incremental validity of sense of coherence, neuroticism, extraversion, and general self-efficacy: longitudinal prediction of substance use frequency and mental health

**DOI:** 10.1186/s12955-016-0412-z

**Published:** 2016-01-14

**Authors:** Dennis Grevenstein, Matthias Bluemke, Henrik Kroeninger-Jungaberle

**Affiliations:** Psychological Institute, University of Heidelberg, Hauptstraße 47-51, 69117 Heidelberg, Germany; Institute of Medical Psychology, Center for Psychosocial Medicine, University Hospital Heidelberg, Bergheimer Str. 20, 69115 Heidelberg, Germany

**Keywords:** Sense of coherence, Neuroticism, Extraversion, General self-efficacy, Incremental validity, Substance use, Psychological distress

## Abstract

**Background:**

Several studies have demonstrated the importance of sense of coherence (SOC), neuroticism (N), extraversion (E), and general self-efficacy (GSE) for health, yet the unique utility of these overlapping constructs remains uncertain. The present research aims at exploring incremental validity when predicting (1) substance use specifically and (2) mental health generally among adolescents.

**Methods:**

A prospective and longitudinal design was used to predict (1) initial substance use nine years into the future and (2) mental health one year and four years into the future. Participants were 318 adolescents (age 14 to 15 at the beginning of the study).

**Results:**

Structural equation modeling revealed (1) that SOC had long-term incremental validity over N, E, and GSE for tobacco use and alcohol use, whereas cannabis use was predicted by E and GSE; and (2) that long-term mental health after four years was only predicted by SOC.

**Conclusions:**

Two studies provide further evidence for the importance of considering salutogenic factors when forecasting mental health and health-related behavior beyond classical constructs such as N, E, and GSE. Differences in criterion validity reveal that SOC cannot be equated with reversed neuroticism.

## Background

Several positive and negative factors on the personality level are supposed to influence mental health and health-related behavior. The proposed factors are often embedded in different theoretical backgrounds. Among the most prominent trait-like constructs are broad measures of personality such as the Big-Five with a focus on neuroticism and extraversion as risk and protective factors [1], sense of coherence as a salutogenic factor [2–4], and constructs based on the experience of one’s own self-regulation attempts such as self-efficacy [5, 6] viz. trait-like general self-efficacy [7, 8]. There is ample empirical evidence that all of these constructs are somehow related to health, yet the unique utility of each one is unclear. The present study contributes to an answer to an unsettled question: Which of the traits shows the highest criterion validity for mental health and substance use outcomes? Both criteria are prime indicators of health during adolescence. The use of psychoactive substances plays an important role in adolescence and is a critical aspect of adolescent health behavior [9, 10]. Not only is current substance use relevant for learning self-regulation and various adverse health outcomes, but also both early and high-frequent consumption levels have been connected to future substance misuse [11].

In the context of developmental tasks [12, 13] it is essential to compare various protective and risk factors not only in a cross-sectional design, but longitudinally in a neck-to-neck comparison. To the extent that any of these constructs demonstrate incremental validity when testing their predictive validity concurrently, one can argue that they cover unique aspects, hence are not merely redundant [14]. On a theoretical level, unique predictive validity can pinpoint the most important factors in aetiopathogenesis and salutogenesis. Thereby, a longitudinal comparison of incremental validity contributes to our understanding of the relative importance—and mutual relationships—of various health-related personality factors.

We will briefly review the personality constructs investigated in the current study, before we discuss a crucial theoretical point: In many cases a strong overlap between these constructs has been shown. Yet, are they redundant indicators of the same latent trait or do some have unique predictive validity?

### Psychological predictors of mental health and substance use

#### Sense of coherence

Health is not just the mere absence of illness and mental disorders. According to Antonovsky’s influential salutogenic approach, health depends on internal resources such as “sense of coherence” (SOC) [2–4]. SOC describes a specific way of viewing life as comprehensible, manageable, and meaningful—especially when critical life events, hardships, and stress add to an individual’s burden. Three major factors underlie SOC: *comprehensibility*, that is, an individual’s perception that situations and events are structured and clear; *manageability*, that is, an individual’s belief that she has the necessary skills to deal with the challenges of life; and *meaningfulness*, that is, an individual’s belief that the demands and challenges of life are worthy of investment and engagement. SOC is one way of looking at several general resistance resources (e.g., resilience, hardiness) [15] that provide an individual with the psychological means to cope with adversities and distress, ultimately nourishing psychological well-being and good mental health.

There are numerous studies that link SOC to positive mental health and health-related behaviors [16]. They range from general psychological well-being [17] to burnout [18], and they encompass depression [19–21] as well as anxiety [22, 23]. With particular regard to substance use, high SOC scores have been shown to predict reduced consumption of tobacco, less intake of alcohol [24], as well as less alcohol-related behavioral problems [25]. The stability of inter-individual differences has been shown before, both for adults [26, 27] and adolescents [28]. For adults, test-retest reliabilities of .78 over one year, .59−.67 over five years, and .54 over ten years have been reported [29]. Hence, SOC appears to be a valuable resource, also from the perspective of public health, if its role in promoting health can be firmly established against the role of competitors.

#### General self-efficacy

Self-efficacy (SE) describes a person’s belief to exhibit control and succeed in a given situation [6, 30]. As an important mediator before intentions can manifest as concrete behavior it plays a central role in health-psychological models [5, 31]. Its positive influence has been evident for some time in the domain of clinical psychology [32], for instance, as an aspect relevant to substance use [33, 34]. SE—commonly understood in terms of a refusal SE, that is, one’s ability to refuse to drink in a tempting situation [34]—plays a role when predicting the use of alcohol [10], tobacco [35], and marijuana [36].

Going beyond specific situations, general self-efficacy (GSE) was conceptualized as people’s SE across a broad range of challenging situations that require effort and perseverance [7, 8]. Though situation-specific SE may be a stronger predictor by accounting for more variance in a specific challenge [6], GSE has the potential to better predict general outcomes, or outcomes in tasks for which an individual has not yet developed expectancies [37]. Finally, it has been shown that task-specific SE and GSE interrelate and influence each other and that GSE builds from more specific SE [38]. GSE most closely matches other trait-like personality constructs examined in this study. Test-retest reliabilities of GSE over one year have amounted to .55 in a sample of adolescent students and .75 for adult teachers [39].

#### Neuroticism

One of the best-known traits, neuroticism (N), is conceptualized as a factor-analytically derived, basic personality trait related to negative affectivity such as anxiety and depression (or in its reversed form: emotional stability). Being part of three-factor-models [40] and five-factor-models [1], there is abundant evidence that N is associated with health-related outcomes. It has been linked to various aspects of mental and physical health [41–46]. Specifically, it predicts alcohol use [47–49] and smoking habits [50, 51].

#### Extraversion

Coming from the same theoretical background, extraversion (E) is conceived as a basic personality trait and characterized by energy, dominance, positive emotionality, and sociability [1, 52]. While the evidence is not as compelling as with N, lower E tends to be associated with social phobia and cluster C personality disorders [53] as well as depression [52, 54]. E has also been linked to increased alcohol consumption: Enhancement drinking (rather than drinking to cope with depression) was predicted by higher levels of E [49, 55]. Likewise, increased tobacco use and earlier onset of adolescent smoking have been longitudinally associated with E [56, 57].

The inter-individual stability of the basic traits over the life course has been documented many times with test-retest correlations ranging between ρ = .46 to ρ = .55 [58–60]. Thus both N and E have a strong a-priori potential for long-term prediction.

### Construct overlap as a contaminant for criterion prediction

Previous research has questioned the distinctiveness of SOC as considerable variance overlap with N has been demonstrated [61–64]. The case has been made that SOC measures N indirectly to the degree that it taps into aspects of emotional stability inadvertently [65]. According to Antonovsky’s [2] theory, however, SOC should not be regarded as a temperamental personality trait like N, rather as a person’s acquired and generalized “orientation to life” in relation to perceiving and controlling the environment for meaningful and appropriate action. Unlike N, for which trait stability and genetic influences have been documented [66], SOC is supposed to be malleable and acquired during adolescence; it develops well into adulthood to finally reach stability around the age of 30 [2]. Based on this purported malleability, it was suggested that SOC could not be a reasonable predictor of health in the long term. Instead, SOC itself might better be explained by personality traits, or it might emerge as a mere correlate of other traits, or appear as a byproduct of mental health, rather than constitute a causal factor for health on its own [14]. Recent accumulating evidence shows that up to 40 % of SOC variance can be explained by Big Five personality traits [67, 68]. However, in Hochwälder’s study only N was consistently related to SOC for both men and women (and E for women) once socioeconomic status was controlled. Hence, it appears that N and E might yield potentially powerful alternative explanations for mental health and drug consumption, which is why we include both of them in our design. These findings question SOC as a theoretically derived construct, because neuroticism has a convincing biological basis [69, 70], including genetic foundations [66, 71].

Empirically, despite its purported malleability and continuous formation across adolescence, SOC is applicable to children already at age 12 [72]. Even though it is expected to be fluctuating at young age, empirical findings suggest that SOC can be quite stable at least over the course of 5 years [27, 73–75].

In terms of theoretical overlap between the constructs, the manageability facet of SOC is reminiscent of general self-efficacy: a person’s belief to succeed and cope with difficulties in life. Given the apparent overlap between these constructs, the present research aims to shed light on the incremental validity of SOC beyond N, E, and GSE when predicting various health-related variables longitudinally. Most notably, it is currently unknown whether adolescents have developed SOC and GSE sufficiently, so that stable individual differences could be used to predict future health-related developments. Going beyond simple cross-sectional correlations, our study is—to our knowledge—the first to investigate the relative merits of these competing risk and protective factors for substance use and mental health in adolescents in a longitudinal design that reaches about a decade into the future, into young adulthood.

### Study overview

The following research is part of a ten-year-longitudinal study of drug use patterns (RISA) conducted in the Rhine-Neckar metropolitan region in the South of Germany between 2003 and 2012. Across 14 data collection events, participants filled in various health-related questionnaires. Using a prospective and longitudinal design, we have investigated two research questions (RQ). In RQ1 we examined incremental validity of salutogenic aspects (SOC) over competing personality factors (N, E, GSE) for the long-term prediction of substance use of alcohol, tobacco, and cannabis from age 15 to 24. RQ2 investigated the incremental validity of SOC at age 15 for lowering the risk of mental health issues 4 years into the future.

## Methods

### Procedure and sample

Participants were 318 students from four different schools aged 14 at the beginning of the study, and aged 24 at the end of the study. Gender was fairly balanced with 164 female (51.57 %) and 154 male (48.43 %) participants. The sample was ethnically diverse. Of all participants, 54.1 % (*n* = 172) were of German nationality, 15.7 % (*n* = 50) did not possess German nationality, while 30.2 % (*n* = 96) did not provide that information. These data are comparable to the official census which denotes 19.3 % of all students in south-west Germany having a migration background [76]. The sample can be characterized as rural or sub-urban with participants living in smaller cities up to 100000 inhabitants. Most participants (65.4 %) grew up in a traditional family, which was defined as living with both biological parents up to the age of 18 years. Level of education was balanced across the three-tier German school system. Informed consent and written permission from legal guardians were obtained. As this was a panel study, there were no specific inclusion or exclusion criteria other than voluntary participation. The study was approved by the ethics committee of the University Hospital Heidelberg (No. 218/2005). The RISA study included 14 data collection events. Up to the age of 16, participants filled out questionnaires every 6 months. From age 17 onwards, surveys were conducted yearly. The data collection was initially carried out in schools by researchers working in the RISA project to ensure independence from teachers, parents, and peers. Participants were divided into smaller groups and data was collected in separate calm rooms to minimize influences of social desirability or peer group pressure. In later years data collection was continued via mail. Individual participants returned filled out questionnaires by mailing them back to the research team. As expected, there was noticeable sample attrition over the course of 9 years. In comparison to participants retained in the study until age 24, participants who dropped out consumed moderately more tobacco at age 15, *M*s = 3.62 vs. 2.58 (*SD*s = 2.72 vs. 2.16), *t*(196.56) = 3.35, *p* = .001, Cohen’s *d* = 0.42 and had slightly higher extraversion at age 15, *M*s = 9.25 vs. 8.30 (*SD*s = 2.54 vs. 2.78), *t*(283) = 2.98, *p* = .003, *d* = 0.36. There were no signs of systematic dropout concerning any other study variable. Finally, sample attrition was comparable to other longitudinal studies concerning the development of adolescents [77].

The present research analyzes data at age 15 (*N* = 286; attrition = 10.1 %), age 16 (*N* = 268; attrition = 15.7 %), age 19 (*N* = 177; attrition = 44.3 %), and age 24 (*N* = 184; attrition = 42.1 %). At age 15, we measured the predictors SOC, N, E, and GSE. Substance use was measured at age 15 and age 24, whereas mental health was measured at age 16 and age 19. To minimize possible bias due to missing data and to reduce the amount of missing cells to be estimated in the SEMs, participants were included for RQ1 if valid data were available for at least one substance at both age 15 and age 24 (*N* = 164); participants for RQ2 (*N* = 162) had to have valid mental health data at both age 16 and age 19. Consequently, the amount of cells that had to be estimated in the SEMs was 5.49 % in RQ1, and none in RQ2.

### Measures

#### SOC-13: sense of coherence

SOC was measured using an abbreviated German 13-item adaptation of the original Orientation to Life questionnaire with 5-point rating scales ranging from 0=“very rarely” to 4=“very often” most of the time [78]. The three distinct SOC facets (comprehensibility, manageability, meaningfulness) were represented by four meaningfulness items (e.g., “Do you have the feeling that you don’t really care about what goes on around you?”), five comprehensibility items (e.g., “Has it happened in the past that you were surprised by the behavior of people whom you thought you knew well?”) and four manageability items (e.g., “Has it happened that people whom you counted on disappointed you?”). Antonovsky [2] stressed the holistic nature of the SOC scale and consequently, a sum score is commonly used. For comparability with an authoritative German version developed by Schumacher and colleagues later [79, 80], scores were rescaled to a 7-point rating scale format using a linear transformation.^1^ In our sample, Cronbach’s Alpha of the scale was .87 at age 15.

#### FPI-R: neuroticism and extraversion

Neuroticism and Extraversion were assessed with the Freiburger Personality Inventory (FPI-R) [81, 82]. The FPI is one of the most prominent German language measures of personality and includes 138 items constituting ten specific subscales, e.g. “irritability” and “social orientation”. From the same item pool, two more general subscales measuring the broader (second order factor) dimensions of Neuroticism and Extraversion (14 items each) according to Eysenck [40] can be additionally composed. Exemplary items are “I ruminate much about my life” (N) or “I like to go out at nights” (E). Participants provided binary answers on whether they deemed items applicable to them or not (1=“true”, 0=“not true”), with negatively keyed questions being recoded afterwards. Affirmative items are summed up to scale scores. At age 15, Alpha amounted to a marginally satisfactory reliability of .64 and a reassuring level of .76 for E and N, respectively. Other scales of the FPI were not considered due to item overlap between the two general scales and the specific scales. Convergent validity of the FPI with other measures of personality was examined in the past and it was shown that the FPI adequately captures the second order factors N and E according to Eysenck [82, 83]. Our own data reflect this as E and N were orthogonal in our sample, which mirrors the original data from Fahrenberg and colleagues.

#### GSE: general self-efficacy

The general self-efficacy scale was developed by Jerusalem and Schwarzer [7, 84]. It comprises ten items such as “If there are challenges, I can find a way to succeed.” Answers were given on 4-point scales (1=“not true”, 2=“rarely true”, 3=“mostly true”, 4=“completely true”). Cronbach’s Alpha was .86 at age 15.

#### SUF: substance use frequency measure

The SUF was adapted from the national survey on drug use among adolescents [85]. It is similar to the brief drug use frequency measure provided by O’Farrell and colleagues [86]. Participants reported on their frequency of use in the past 6 months for tobacco, alcohol, and cannabis. Hence, substance use frequency was measured using single item questions (“*How often have you consumed this substance in the last 6 months?”*) on 7-point frequency scales (1=“not used in last 6 months”, 2=“1–2 times in the last 6 months”, 3=“3–5 times in the last 6 months”, 4=“1–3 times a month”, 5=“1–2 times a week”, 6=“several times a week”, 7=“several times a day”).

#### SCL-90-R: mental health

The Symptom Checklist-90-R (SCL-90-R) [87] asks for a wide range of psychological and psychopathological symptoms. We used the revised German version [88]. Ninety items yield nine different subscale scores and three global scores. In our sample, reliabilities of the subscales ranged from Alpha = .75 to .87 at age 16 and from Alpha = .72 to .89 at age 19. The Global Severity Index (GSI), a measure of general psychological distress computed as the mean over all the SCL-90-R items, formed our variable of interest with an Alpha of .98 and .97 at age 16 and age 19 respectively.

### Statistical analysis

Using SPSS 21 [89] for descriptives and Mplus 7.11 [90] for Structural Equation Modeling (SEM), we examined the relationships between the target variables. Although similar to regression analysis, SEM allows us to model the longitudinal influences of variables over time, while controlling for mutual influence and other confounding factors [91, 92]. For longitudinal predictions, this also enables us to control for initial levels of substance use or psychological distress. As all variables can be modeled simultaneously, the incremental validity of SOC, N, E, and GSE becomes evident in significant predictive paths from the variable of interest to the criterion variable, reflecting semi-partial regression weights. Full Information Maximum Likelihood (ML) was used for the parameter estimation and the handling of missing data.^2^

To estimate whether the model accurately represented the observed data, goodness-of-fit was evaluated by (1) the—ideally non-significant—*χ*^2^ test [93] and as low as possible a *χ*^2^/*df* ratio, ideally as low as 2 [94]; (2) the comparative fit index (CFI) with values of .90/.95 and above indicating appropriate/good model fit [95, 96]; (3) the root mean square error of approximation (RMSEA) with values of .00–.05/.06–.08/.09–.10 indicating good/reasonable/poor model fit [97]; and (4) the standardized root mean square residual (SRMR) with values less than .08 considered to reflect good fit [96].

## Results

### Descriptive data analysis

Table 1 presents means and standard deviations at different points in time, including the cross-sectional analysis of gender differences. Although 15-year old male adolescents reported higher GSE, higher SOC, and lower N than female adolescents, they also consumed alcohol and cannabis more frequently. Existing gender differences amplified across the following 9 years. At age 24 men used alcohol and cannabis significantly more often than women, and the respective effect sizes increased by factor two to three. Paired *t*-tests indicated that substance use of tobacco (*t*(140) = 5.29, *p* < .001), alcohol (*t*(158) = 10.99, *p* < .001), and cannabis (*t*(109) = 2.63, *p* = .01) increased from age 15 to age 24. Inter-individual differences in tobacco use were only moderately stable at *r* = .41, followed by alcohol (*r* = .35) and cannabis use (*r* = .20). These results indicate that we were just looking at the onset of substance use and stable patterns of use had not yet emerged at this early age. With regard to psychological distress, no mean differences emerged comparing ages 16 and 19 (*t*(161) = 0.96, *p* = .34) and inter-individual differences were quite stable at *r* = .57.

**Table 1:** Sample characteristics, significance tests, and effect sizes (Cohen’s d) across Study 1 and 2 at ages 15, 16, 19, and 24. Sample sizes reflect the maximum number of participants. Different degrees of freedom indicate missing data and/or unequal variances

	Total (*N* = 318) *M* (*SD*)	Men (*n* = 154) *M* (*SD*)	Women (*n* = 164) *M* (*SD*)	*t (df)*	*p*	*d*
Sense of coherence (SOC) 15	63.55 (10.89)	65.58 (10.16)	61.82 (11.22)	2.91 (276)	.004	0.34^***^
Neuroticism (N) 15	6.39 (3.30)	5.80 (3.29)	6.91 (3.22)	*−*2.87 (284)	.004	*−*0.34^***^
Extraversion (E) 15	8.68 (2.72)	8.89 (2.81)	8.49 (2.63)	1.23 (284)	.220	0.15
General Self−efficacy (GSE) 15	2.86 (0.46)	2.92 (0.45)	2.80 (0.46)	2.20 (283)	.028	0.26^**^
Tobacco use 15	2.99 (2.45)	2.97 (2.50)	3.01 (2.41)	*−*0.13 (274)	.900	*−*0.02
Alcohol use 15	2.78 (1.38)	2.92 (1.49)	2.64 (1.27)	1.68 (274)	.094	0.20^*^
Cannabis use 15	1.37 (1.00)	1.57 (1.26)	1.19 (0.63)	3.06 (184.83)	.003	0.38^***^
Tobacco use 24	3.90 (2.70)	4.01 (2.75)	3.79 (2.68)	0.49 (148)	.622	0.08
Alcohol use 24	3.95 (1.26)	4.35 (1.16)	3.57 (1.23)	4.28 (168)	< .001	0.65^****^
Cannabis use 24	1.63 (1.15)	2.00 (1.37)	1.24 (0.71)	3.81 (89.28)	< .001	0.70^****^
SCL90-R GSI 16	0.46 (0.43)	0.45 (0.43)	0.47 (0.43)	*−*0.44 (266)	.664	*−*0.05
SCL90-R GSI 19	0.42 (0.40)	0.41 (0.33)	0.43 (0.44)	*−*0.37 (175)	.713	*−*0.05

Men and women differ significantly at ^*^ *p* < 0.10, ^**^ *p* < 0.05, ^***^ *p* < .01, ^****^ *p* < .001

### Correlational analysis

As expected, the overlap between the constructs was evident in substantial relations between SOC, N, and GSE as well as E and GSE (cf. Table 2). Mirroring previously reported findings, N and SOC shared about 41 % of their variance [67]. The consumption of various substances was moderately related.

**Table 2:** Inter-correlations of predictors (age 15; Study 1 & 2), substance use (age 15 & 24; Study 1), and mental health (age 16 & 19; Study 2)

	SOC 15	N 15	E 15	GSE 15	TOB 15	ALC 15	CAN 15	TOB 24	ALC 24	CAN 24	SCL 16
Sense of coherence (SOC) 15	-										
Neuroticism (N) 15	−.64^****^	-									
Extraversion (E) 15	−.03	.05	-								
General self-efficacy (GSE) 15	.47^****^	−.26^****^	.32^****^	-							
Tobacco use (TOB) 15	−.22^****^	.19^***^	.22^****^	−.04	-						
Alcohol use (ALC) 15	−.10^*^	.13^**^	.29^****^	.03	.50^****^	-					
Cannabis use (CAN) 15	−.07	.11^*^	.17^***^	−.02	.46^****^	.40^****^	-				
Tobacco use (TOB) 24	−.31^****^	.21^**^	.18^**^	−.09	.41^****^	.22^**^	.15^*^	-			
Alcohol use (ALC) 24	−.15^*^	.01	.08	−.04	−.02	.35^****^	.11	.19^**^	-		
Cannabis use (CAN) 24	−.11	.07	.21^**^	−.12	.10	.05	.20^**^	.38^****^	.31^***^	-	
Mental health (SCL) 16	−.49^****^	.43^****^	.09	−.13^**^	.22^****^	.18^***^	.04	.21^**^	.11	.07	-
Mental health (SCL) 19	−.39^****^	.32^****^	.03	−.18^**^	.12	.07	.04	.10	.01	.05	.57^****^

Bivariate *N*s range between 91 and 286; ^*^ *p* < 0.10, ^**^ *p* < .05, ^***^ *p* < .01, ^****^ *p* < .001

### Research question 1: substance use

#### Structural equation modeling

SEM allowed us to track individual developmental changes in substance use while estimating the predictive validity of various protective and risk factors. Our model included SOC, N, E, and GSE at age 15 as exogenous variables, which predicted the endogenous variables alcohol, tobacco, and cannabis use frequency at age 15 (cross-sectionally) and at age 24 (longitudinally). Longitudinal paths thus represent semi-partial regression weights and denote the predictive power of personality, essentially controlling for initial levels of substance use. The paths from each substance at age 15 to the same substance at age 24 reflected autocorrelations.

At every point in time, the consumption frequencies were allowed to covary, as were the predictors SOC, N, E, and GSE. In a first step, an unrestricted model examined the incremental validity of the target variables over and beyond others. In a second step, we retained only the significant (or marginally significant) paths. The more restricted model gained degrees of freedom for stricter model testing and provided a more comprehensible view on the relative usefulness of the constructs. For transparency we present both models as this approach is comparable to hierarchical regression analysis, so the absolute strength of the various paths depends on additional predictors in the model.

**Fig. 1:**
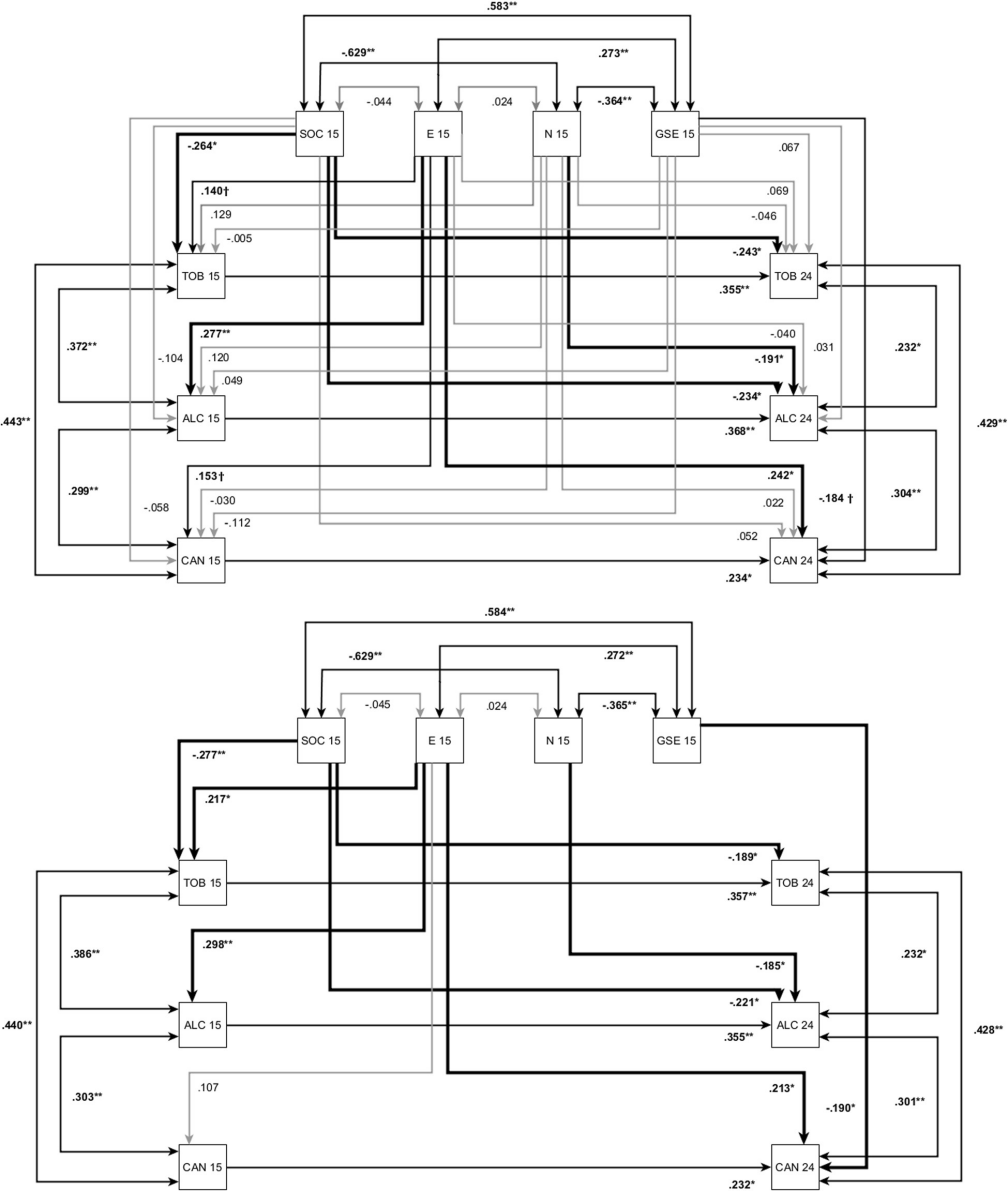
Unrestricted SEM (Fig. 1a, top panel) and restricted SEM (Fig. 1b, lower panel) with standardized regression estimates for sense of coherence (SOC), neuroticism (N), extraversion (E), self-efficacy (GSE) at age 15 and consumption of tobacco (TOB), alcohol (ALC) and cannabis (CAN) at age 15 and age 24 (T14) (time interval = 9 years) with *N* = 164. Paths are significant at †*p* < .10, ^*^ *p* < .05, ^**^ *p* < .01, and ^***^ *p* < .001

The top panel of Fig. 1 shows the unrestricted model with estimated standardized path coefficients. All goodness-of-fit indices showed good to excellent model fit, *χ*^2^(6) = 11.01, *χ*^2^/*df* = 1.83, *p* = .09, RMSEA = .071, CFI = .975, SRMR = .028. With regard to cross-sectional validity at age 15, tobacco use was significantly associated only with SOC, but not with N, E, or GSE. By contrast, alcohol use was significantly related only to E, but not to SOC, N, or GSE. Cannabis use could only be marginally predicted by E, but alternative predictors did not fare any better. The pattern showed that N was least likely to explain why young people at age 15 consumed any type of psychoactive substances; evidently, relief from negative affectivity was not the driving force. By comparison, E seemed to play a bigger role in this regard. Furthermore, salutogenic resources captured by SOC constituted a relevant factor, though they are thought to develop just during adolescence.

The longitudinal part of the model showed that, unsurprisingly, substance use at age 15 predisposed individuals to their drinking or smoking habits nine years later as 24-year-old adults. The incremental validity of personality constructs at age 15, while controlling for these autocorrelations, was even more revealing. Previous substance consumption, but also SOC, predicted tobacco use at age 24; hence SOC was not only associated with reduced smoking habits at age 15, higher SOC at age 15 was also associated with lower the risk of smoking at age 24, even controlling for the initial level of substance use at age 15. Future alcohol use was incrementally predicted by SOC too, and N exerted some influence independently of SOC. Up to here, both findings speak to SOC as a preventive factor for excessive substance use not reflected by any other construct. By contrast cannabis consumption at age 24 was neither predicted by SOC or N, only by E and marginally by GSE. At least in our sample cannabis consumption is unlikely to reflect escapism from life, but rather sociability and self-reliance.

The restricted model (Fig. 1, lower panel) reflects the inclusion of (at least marginally) significant paths from the unrestricted model. Model fit increased as compared to the unrestricted model, *χ*^2^(21) = 24.72, *p* = .26, RMSEA = .033, CFI = .981, SRMR = .051. The only differences emerging between the two models were that (1) the formerly marginally significant path from E to tobacco use at age 15 became significant; (2) cannabis use at age 15 was not significantly related to E any longer; and (3) the formerly marginally significant path from GSE to cannabis use at age 24 turned significant. All in all, the paths reflected the fact that every construct was somehow related to substance use, albeit in specific ways for the different substances.

### Research question 2: mental health

#### Structural equation modeling

**Fig. 2:**
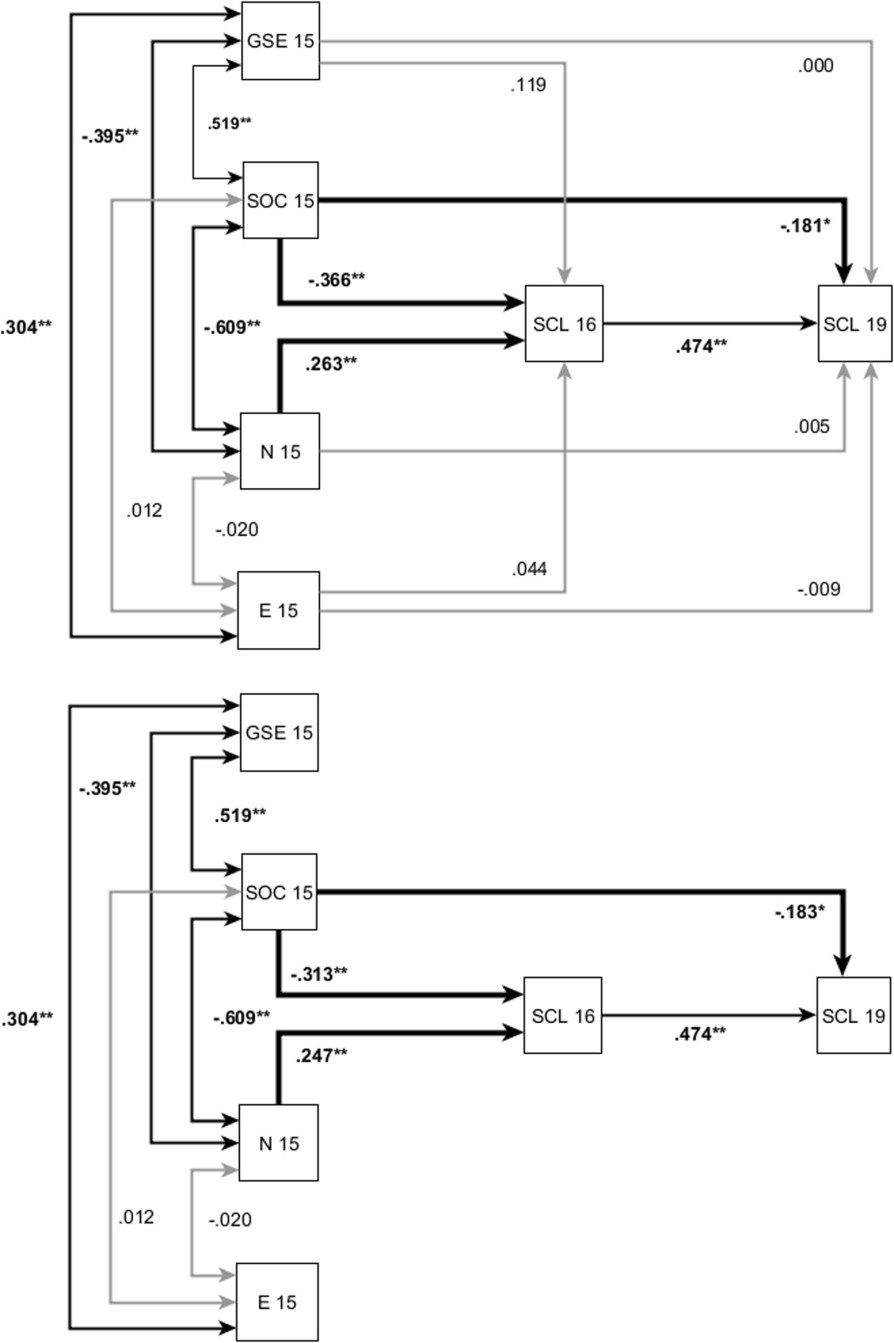
Unrestricted SEM (top panel) and restricted SEM (lower panel) with standardized regression estimates for sense of coherence (SOC), neuroticism (N), extraversion (E), self-efficacy (GSE) at age 15 and mental health (SCL) age 16 and age 24 with *N* = 162. Paths are significant at ^*^ *p* < .05, ^**^ *p* < .01, and ^***^ *p* < .001

RQ2 focused on general mental health outcomes. The SEM modeled three time points (age 15, 16, and 19). SOC, N, E and GSE at age 15 were used to predict psychological symptoms at age 16 and 19. Psychological symptoms at age 19 were additionally predicted by symptoms at age 16 (autoregressive process). The model included cross-sectional correlations among all the predictor variables at age 15. The first step was to evaluate incremental validity of the exogenous variables in a full model. This unrestricted model is just identified, as all the variables are connected to each other, leaving no spare degrees of freedom. Even though we can examine incremental validity, similar to a hierarchical multiple regression analysis, model fit cannot be computed. (In a subsequent step, we restricted the model such that all paths not marginally significant at least were discarded.) The top panel of Fig. 2 displays the standardized estimates for the unrestricted model. Psychological symptoms (SCL) at age 16 could be significantly predicted by both SOC and N, with SOC being a somewhat stronger predictor. Furthermore, SCL at age 19 could be significantly predicted by SCL at age 16, yet most importantly by SOC and only by SOC.

This pattern did not change in the restricted model (cf. lower part of Fig. 2). Removing several paths (constraining them to zero) gained us some degrees of freedom, so that model fit could then be estimated. The model fitted the data extremely well, *χ*^2^(5) = 3.36, *p* = .44, RMSEA = .000, CFI = 1.000, SRMR = .025.^3^ While SOC had only a slight predictive advantage over N for SCL at age 16, SOC clearly was the exclusive predictor of future development of mental health at age 19.

### Additional analyzes

We conducted additional analyses to investigate any possible influences of covariates. The models were recomputed while controlling for gender, family setting, and the amount of pocket money as reported by participants at age 15 as a proxy for their socio-economic status. The addition of any of the covariates did not substantially alter the paths in the models or affect any of the conclusions regarding incremental validity. Model fit, however, decreased noticeably, even beyond conventionally accepted limits. Thus adding covariates was hardly reasonable.

The comparatively lower internal consistency of N and E might be of concern to some readers, as the lower criterion validity might be attributed to a lack of reliability. We therefore modeled N and E as latent variables in the SEM, rather than using sum scores. This approach enables us to estimate the paths in the model free from measurement error. Still, the associations of N or E with the outcomes did not improve. Failing conventional rules of thumb regarding sample size, we refrained from modeling and reporting analyses based on latent variables.

## Discussion

The present study explored the incremental validity of sense of coherence (SOC), neuroticism (N), extraversion (E), and general self-efficacy (GSE) when longitudinally predicting health-related outcomes. While not denying that, pragmatically, the functional value of constructs may differ depending on the criteria at hand, our results showed that for several highly relevant variables SOC contributed uniquely or even had a clear-cut advantage over N, E, and GSE, especially in the long run.

We investigated substance use of tobacco, alcohol, and cannabis. Looking at the earliest point in time, E was the most important predictor for use of any of the substances. Reflecting the extant literature, we interpret this finding as follows: Extraverted adolescents may be more likely to be exposed to social settings in which tobacco, alcohol, and cannabis are accessible. These findings reflect that substance use, just like other risky youth behavior, is heavily influenced by peers [98], and extraverted adolescents are more prone to find themselves in social situations where the presence of peers may promote substance use [99]. Tobacco use was predicted by SOC, hinting at SOC as a valuable protective factor against harmful tobacco use already at a relatively young age. Looking at the last point in time, SOC still forecast tobacco use, but not any other construct. Alcohol use was equally well predicted by SOC and N, with a small numerical advantage for SOC. Various personality aspects were associated with future use of alcohol and tobacco even when controlling for previous differences in behavioral tendencies, yet in sum SOC had the highest incremental validity.

Cannabis use, however, differed from the use of the other psychoactive substances. No relationship with either SOC or N was found; instead, cannabis use was predicted by E and GSE. Young adults who scored higher in E and lower in GSE were more likely to use cannabis. This was the only case where GSE had incremental validity over SOC and N. Although our data do not allow evaluating specific situations, one might tentatively conclude that cannabis use was stimulated by social situations as indicated by its relationship with E. The importance of GSE might then reflect *refusal* self-efficacy in social settings, rather than *use* self-efficacy [34], in other words, a person’s capacity to decline any offer to use drugs in a tempting situation. This interpretation is augmented by the fact that, at the time of the study, cannabis use was illicit in Germany—and dealing still is—yet mere private consumption of small amounts of cannabis is not any longer punishable by German law. Taken together, our results indicate that the use of different substances if diversely associated with different aspects of personality. This may in part be responsible for notable differences in typical consumption patterns for different substances [100].

In the second part of the present research we examined the relationships among SOC, N, E, GSE, and mental health. Both SOC and N at age 15 predicted psychological symptoms at age 16, yet only SOC was able to project mental health even further into the future. As neither E nor GSE showed any signs of incremental validity, unspecific mental health seemed strongly determined by emotional (in)stability as measured by SOC and N. Again, SOC emerged as a superior predictor for mental health in the long term. While this may come as a surprise to some, SOC’s advantage is well in line with recent findings. Gale et al. [42] observed that N had a long-term effect on mental well-being. Most of its impact was mediated through people’s susceptibility to psychological distress and physical health problems. At this point, an inclusive interpretation would suggest that SOC buffers against psychological distress by facilitating specific coping styles that contribute to people’s health, even in the face of adverse events.

### Limitations

As a potential shortcoming of our research might count that N and E were not measured with classic inventories such as the NEO-PI-R, NEO-FFI [1], or EPQ [101]. Still, the measures we used are frequently used in German research contexts, and with good success [82, 102]. Especially in the context of substance use, personality factors beyond E and N, namely conscientiousness (C), are of great importance. Then again, a recent study confirmed that SOC was most dominantly related to N, and that N was the strongest competitor for criterion validity [67]. In fact, SOC could be shown to possess substantial incremental validity over all the Big Five when cross-sectionally predicting mental health, satisfaction with life, and personal distress, even when using an up-to-date inventory of the Big Five personality traits, providing higher reliability (Alpha) than the older FPI-R used in the present research. We conclude that the present research closely converges with independent findings. Attributing the currently observed incremental validity of SOC to a lack of reliability on the side of other predictors is unwarranted.

We found that participants who dropped out of the study reported more extraversion and more tobacco use at age 15. This may hint at aspects of selective dropout. For our core variables of interests however, there were no hints at systematic sample attrition. One disadvantage of our study design is that the SCL-90-R, our measure of psychological distress, was not available at ages 15 and 24, the starting and end points for the present analyses. Nonetheless, we can investigate development of mental health from age 16 to age 19. Socioeconomic status was assessed using the amount of pocket money available to the adolescents at age 15. This can only be a proxy variable, yet it is obvious that adolescents have to pay for their substance use with their own money. However, we do not expect this to be a perfectly reliable indicator of the multitude of potential influences associated with socioeconomic status [103]. Finally, our study was conducted in Germany. Cultural issues may therefore affect the generalization of our findings to other contexts. This may be especially true for substance use and misuse, which is highly affected by political issues and varies across different countries [104].

Future research should run similar research designs while using other instruments and even expand the idea to other constructs such as hardiness [105] or dispositional optimism [106]. The latter constructs are expected to improve one’s health status, too, whereas their distinctiveness seems doubtful. With regard to the development of adolescents further research could shed more light on what personality factors are specifically influential and important. This might then better reflect the diversity of adolescent development paths.

## Conclusions

There is a marketplace full of different constructs that may have overlapping, but also unique characteristics. Many come from different theoretical backgrounds, and yet they are rarely tried against each other. For the present research we chose mental health and health-related behavior and investigated incremental validity, tackling the question which construct is capable to predict them best. In many cases SOC had incremental validity over N, E, and GSE. Consequently, we have to refute earlier claims that SOC is merely measuring other traits in disguise [14, 61]. The oft-documented high correlation with neuroticism also emerged in our data [68]. Nonetheless our results highlight that SOC is not just emotional stability or a simply byproduct of health itself. SOC has proven to be a valuable predictor longitudinally.

From a health psychology point of view, this backs up SOC as a genuine construct relevant for health psychology. From a personality psychology point of view, rather than merely insinuating that SOC is an indirect measure of well-established traits, SOC has a right to its own and can coexist next to classic traits. Although some have argued that there would be little additional value beyond the shared variance of a common core among N, E, generalized SE, and locus of control [107], the same authors advised that research should focus on demonstrating unique variance beyond a common core—by using external validation criteria. In following their recommendations, we presented evidence that SOC indeed captures variance over and beyond competing personality constructs, and therefore clearly reflects personality aspects beyond mere emotional stability. Our findings are all the more remarkable as we used the abbreviated (13-item) SOC scale. Strikingly, a brief questionnaire for a psychometrically relatively heterogeneous construct predicted the chosen criteria effectively.

Hardly ever is there universal influence of any construct on all chosen criteria at all the time. Just like mental health, substance use during adolescence is multifactorially determined, not exclusively driven by resilience or emotional stability. The onset of substance use, which is a major factor of risky consumption patterns later in life [11], may depend more on social factors than on individual differences in resilience levels. The present design does not allow inferences about motivations underlying any observed substance use, because the mere frequency of use cannot be equated with problematic consumption. In many ways substance use is an age-specific and transitional phenomenon, partly reflecting age-appropriate, normative behavior.

Despite SOC having an advantage over competing constructs, the psychometric limits of the SOC scale come at a price. It is unknown which facets of SOC are likely to be responsible for the outcomes. At the moment any of the SOC-dimensions (comprehensibility, manageability, meaningfulness) might be the driving force behind predictive validity. Despite recommendations not to use subscale scores, a three-factor structure of SOC has been found to best represent the 29-item SOC scale but not necessarily the 13-item version [80, 108]. Future studies might compare subscale scores from the unabbreviated 29-item scale to other constructs, if only to further elucidate what exactly separates SOC from similar constructs. Such research will provide more theoretical insight into how a general “orientation to life” is different from fundamental personality traits—to shed more light on how salutogenesis differs from pathogenesis.

### Ethics approval and consent to participate

All procedures performed in studies involving human participants were in accordance with the ethical standards of the institutional and/or national research committee and with the 1964 Helsinki declaration and its later amendments or comparable ethical standards. Informed consent was obtained from all individual participants included in the study. The study was approved by the ethics committee of the University Hospital Heidelberg (No. 218/2005).

## Footnotes

1 The rescaled values summed up to *M* = 65.58 (*SD* = 10.16) for men and *M* = 61.82 (*SD* = 11.22) for women. They resemble the norms published by Schumacher et al. [79] who reported a SOC sum score of *M* = 67.31 (*SD* = 12.09) for men and *M* = 64.52 (*SD* = 11.61) for women spanning a wider age range from 18 to 40.

2 Inspection with robust chi-squares and standard errors (MLR; Yuan & Bentler, 1998) showed that non-normal data did not severely affect the outcome or interpretation. Nonetheless, we chose the typical ML estimator as non-normal distributions of substance use and mental health represented the most important part of the variance that we explicitly wanted to incorporate in the model and predict.

3 If *χ*^2^ is less than *df*, which is the expected *χ*^2^ in the population, then RMSEA is set at zero, indicating a perfectly specified model. Similarly, if CFI is greater than one, it is set at one and if less than zero, it is set at zero (see http://davidakenny.net/cm/fit.htm).
